# Vitamin D deficiency in pregnancy – a failure of public health policy?

**DOI:** 10.1186/1753-6561-6-S4-P9

**Published:** 2012-07-09

**Authors:** S Sivalokanathan, T McAree, B Jacobs, T Manickavasagar, L Brennan, P Bassett, S Rainbow, M Blair

**Affiliations:** 1 Imperial College London, UK; 2 North West London Hospital NHS Trust, UK; 3 RNOH NHS Trust, Middlesex, UK

## Introduction

In order to understand the extent of serum vitamin D deficiency we measured vitamin D levels in an unselected multi-ethnic population of pregnant women. We report the prevalence of insufficiency and deficiency, explore risk factors and discuss the public health implications. This report may be the first of its kind.

## Methods

Sample women with sufficient stored serum were randomly selected from among all women who had delivered in year 2008/09. Serum vitamin D levels were determined using liquid chromatography coupled to tandem mass spectrometry). Vitamin D levels were analyzed with respect to ethnicity (as marker for skin tone), calendar quartile, body mass index trimester and parity. Deficiency was defined as < 25 nmol/L, insufficiency 25 - 75 nmol/L, and adequacy > 75 nmol/L.

## Results

Three hundred and forty six women were included and represented the total population in terms of skin tone, quartile, BMI, gestation, and parity. Overall, 18% (95% CI: 15% to 23%) of sample women had adequate vitamin D levels; 36% were deficient, 45% insufficient. Among women with dark skin, only 8% (95% CI: 5% to 12%) had adequate levels compared to 43% (95% CI: 33% to 53%) of those with light skin. Obese women were found have significantly lower Vitamin D levels than non-obese women.

## Conclusions

Vitamin D deficiency and insufficiency are prevalent year round among pregnant women in northwest London, especially those with darker skin. Existing supplementation guidelines should be supported however; other measures are required to improve status among all women.

